# Application of Vibrational Spectroscopies in the Qualitative Analysis of Gingival Crevicular Fluid and Periodontal Ligament during Orthodontic Tooth Movement

**DOI:** 10.3390/jcm10071405

**Published:** 2021-04-01

**Authors:** Fabrizia d’Apuzzo, Ludovica Nucci, Ines Delfino, Marianna Portaccio, Giuseppe Minervini, Gaetano Isola, Ismene Serino, Carlo Camerlingo, Maria Lepore

**Affiliations:** 1 Multidisciplinary Department of Medical-Surgical and Dental Specialties, University of Campania Luigi Vanvitelli, 80138 Napoli, Italy; ludortho@gmail.com (L.N.); giuseppe.minervini@unicampania.it (G.M.); 2 Department of Ecological and Biological Sciences, University of Tuscia, 01100 Viterbo, Italy; delfino@unitus.it; 3 Department of Experimental Medicine, University of Campania Luigi Vanvitelli, 80138 Napoli, Italy; mariannabiancaemanuela.portaccio@unicampania.it (M.P.); ismene.serino@unicampania.it (I.S.); maria.lepore@unicampania.it (M.L.); 4 Department of General Surgery and Medical-Surgical Specialties, University of Catania, 95124 Catania, Italy; gaetanoisola@gmail.com; 5 CNR-SPIN, SuPerconductivity and Other INnovative Materials and Devices Institute, 80078 Pozzuoli, Italy; carlo.camerlingo@spin.cnr.it

**Keywords:** vibrational spectroscopies, Raman spectroscopies, infrared spectroscopies, orthodontic tooth movement, gingival crevicular fluid, periodontal ligament

## Abstract

Optical vibrational techniques show a high potentiality in many biomedical fields for their characteristics of high sensitivity in revealing detailed information on composition, structure, and molecular interaction with reduced analysis time. In the last years, we have used these techniques for investigating gingival crevicular fluid (GCF) and periodontal ligament (PDL) during orthodontic tooth treatment. The analysis with Raman and infrared signals of GCF and PDL samples highlighted that different days of orthodontic force application causes modifications in the molecular secondary structure at specific wavenumbers related to the Amide I, Amide III, CH deformation, and CH_3_/CH_2_. In the present review, we report the most relevant results and a brief description of the experimental techniques and data analysis procedure in order to evidence that the vibrational spectroscopies could be a potential useful tool for an immediate monitoring of the individual patient’s response to the orthodontic tooth movement, aiming to more personalized treatment reducing any side effects.

## 1. Introduction

Orthodontic tooth movement (OTM) is related to remodeling of alveolar bone and periodontal ligament (PDL) with several macroscopic and microscopic biological changes. These events lead to structural modifications of the periodontal ligament (PDL), as well as an increased flow and composition changes of the gingival crevicular fluid (GCF) in the periodontal sulcus. The PDL is a complex, neurovascular, cellular, and connective tissue interposed between the tooth root and the alveolar bone, mainly composed of collagen fibers, whereas the GCF derives from the epithelium lining of the gingival sulcus, thus its characteristics depend on the specific site from which it is collected. Orthodontic force application generates a regular process that is basically characterized by bone deposition at sites of tension and bone resorption on the pressure site upsetting the homeostatic environment of periodontal tissues and locally altering the blood flow and electrochemical conditions. These modifications lead to the generation and propagation of signaling chemical cascades and associated tissue remodeling by delineating biochemical and cellular reactions occurring in tissues and fluids around the teeth [1,2,3]. In the literature, several in vivo and in vitro studies, both in animal and human samples, investigated the structure and molecular patterns of the PDL and GCF during orthodontic treatment through different analytical methodologies (i.e., immunohistochemistry, immunocytochemistry, confocal laser microscopy, gene arrays real-time PCR (Polymerase Chain Reaction), transmission electron-microscopy, microcomputed tomography) [4,5,6,7,8,9,10]. However, these methods are time-consuming and labor-intensive, requiring complex procedures to prepare the samples. In the last years, some optical vibrational techniques as micro-Raman Spectroscopy (μ-RS), Surface-Enhanced Raman Spectroscopy (SERS), and Fourier Transform-Infrared (FT-IR) [11,12,13,14] were used for this purpose. Vibrational spectroscopy is the collective term used to describe analytical techniques that measure vibrational energy levels associated with the chemical bonds. The spectra obtained from these analyses are considered like a fingerprint containing signals from the functional groups in the sample. In particular, Raman techniques measure the characteristics Raman emission induced from molecules under monochromatic laser irradiation while IR measures the light absorption by specific molecules using a broadband light source [15]. Raman Spectroscopies (RS) and FT-IR are complementary techniques because the related bands are due to nonpolar and polar functional groups, respectively, thus they are both needed to detect complete biochemical information of the samples. For these reasons, the vibrational spectroscopies have been utilized in an enormous number of fundamental and applied research fields. In the biomedical fields, these techniques show high sensitivity in revealing detailed information on molecular composition, structure, and interactions with non-destructive sampling and reduced analysis time in comparison to other conventional methodologies. In particular, biofluids are ideal samples for routinely clinical assessment with vibrational spectroscopies being easily accessible through minimally invasive collection methods and repeatedly available for monitoring disease progression or therapeutic response [14,15,16,17,18,19,20,21,22]. Vibrational spectroscopies are thus convenient and reliable techniques in order to obtain an overall biochemical characterization of the main tissue and biofluids involved in the orthodontic tooth movement to properly monitor their microscopic changes during treatment. A comprehensive evaluation of the novel use of these techniques in Orthodontics may provide a wider vision of recent outcomes and future research both for diagnosis and monitoring strategies in this field. In the last years, we devoted great attention to the use of vibrational spectroscopies for monitoring OTM in young and adult patients and this review aims to revise the studies which used these vibrational spectroscopies for the qualitative analysis of PDL and GCF samples collected during the initial phase of OTM in young and adult patients.

## 2. Materials and Methods

### 2.1. Study Selection

To retrieve lists of potential articles to be included in the review, the search strategy included the following databases to December 2020: PubMed, PubMed Central, National Library of Medicine’s Medline, Embase, Cochrane Central Register of Controlled Clinical Trials, Web of Knowledge, Scopus, Google Scholar, Latin American and Caribbean Health Sciences Literature (LILACs). Abstracts, and presentations from international orthodontic meetings were evaluated. To identify relevant records, the search encompassed an effective combination of Medical Subject Headings (MeSH) terms and free-text terms including ‘vibrational spectroscopies’, ‘Raman spectroscopy’, micro-Raman spectroscopy’, ‘infrared spectroscopy’, ‘Fourier-Transform infrared spectroscopy’, AND ‘tooth movement’, ‘orthodontics’, ‘orthodontic force application’, ‘orthodontic treatment’, AND ‘gingival crevicular fluid’, ‘periodontal ligament’.

Title and abstract screening were performed to select articles for full-text retrieval by two reviewers. An initial screening of titles and abstracts against the inclusion criteria was performed to identify potentially relevant papers followed by a screening of the full possibly relevant papers. Duplicate articles were removed, and the studies were selected for inclusion independently by both authors. The concordance percentage between the two reviewers was less than 3% and any doubts or disagreements were resolved after discussion.

A total of seven relevant publications including analysis of human GCF and PDL subjected to orthodontic forces with vibrational spectroscopies were selected. The results reported in these publications (see Table 1) are revised in the present paper. In Table 1 information about first authors and year of publication, analytical vibrational techniques, type of sample collected, duration of orthodontic tooth movement and characteristics of subjects are indicated [23,24,25,26,27,28,29].

### 2.2. Sample Selection

**Figure 1:**
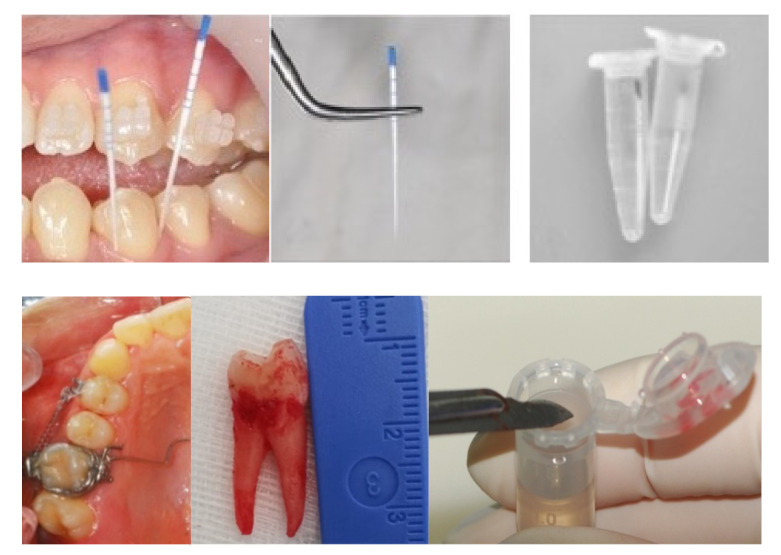
Collection of GCF (line above) and PDL (line below) samples. (Photographs taken at the Orthodontic Program of the University of Campania *Luigi Vanvitelli*).

In the studies here considered, the patients were selected according to the following criteria: (1) young subjects, between 11 and 18 years, and adults over 18 years of age with dental malocclusion needing orthodontic treatment; (2) patients treated with any type of orthodontic force application through fixed appliances; (3) patients without treatment were assumed as controls. GCF and PDL samples were collected from these patients at different time intervals of OTM (Figure 1). The GCF was collected from each patient before bracket bonding (T0), and after 1 or 2 (T1), 7 (T2), and 14 or 28 (T3) days of treatment [24,25,26,27]. As showed in Figure 1, paper points were inserted into the gingival crevice for about 30 s, then placed into Eppendorf PCR Tubes and stored in a −80 °C refrigerator before analysis. Specifically, for μ-RS measurements, the paper points were directly examined without any other treatment [25,26], whereas for SERS and FT-IR measurements, 10 μL of distilled water was added in the tubes that were vortexed and then centrifuged [26,27].

The PDL was scarified from the root of extracted premolars using a one-way lancet after surgery, immediately fixed in 4% paraformaldehyde and stored in ethanol solutions until analysis (Figure 1) [23,27].

### 2.3. Experimental Techniques

#### 2.3.1. Raman Spectroscopy

Raman spectroscopy is considered an effective tool to determine the structure and the chemical composition of materials considering the vibrations of molecules. This technique enables acquiring vibrational spectra of samples by analyzing scattered light due to monochromatic laser excitation [12]. When the laser light beam hits the tissue, it is partly absorbed and partly transmitted or reflected by the sample at the same wavelength of the incident light. Besides, there is a little component that has a wavelength slightly differing from the incident one of a quantity reflecting the typical vibrational modes of the analyzed substance. In biological tissues, the molecular contribution to Raman scattering is mainly related to vibrational transitions which force a molecule to make a transition from a state of vibrational energy E1 to another state E2 (see Reference [30] and References [2,3,4,5] therein). Absorption by atoms or molecules occurs only if the difference in energy ΔE is less than or equal to the quantum photon energy hυ: ΔE = E2 − E1 ≤ hυ, where υ is the light frequency and h the Planck constant.

**Figure 2:**
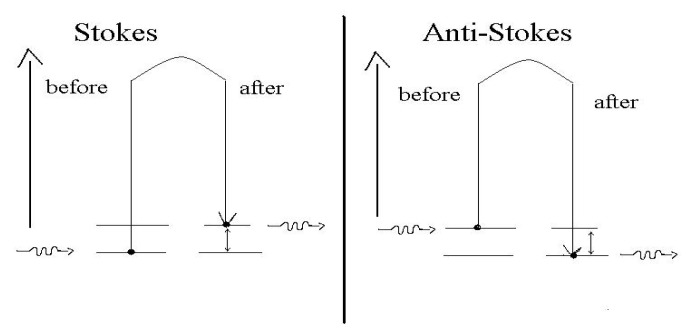
Stokes and Anti-Stokes mechanisms of the Raman scattering.

As shown in Figure 2, an incident photon collides with a molecule that can be either in the fundamental state or in an excited vibrational state. If the impact is of an elastic type, the diffused photon has exactly the same energy and wavelength of that incident (scattering Rayleigh) not altering the energy state of the molecule. On the other hand, if the impact is inelastic (Raman scattering), the diffused photon may have less energy (and consequently less frequency and longer wavelength) or greater energy (higher frequency and shorter wavelength) than the incident photon. The scattered photon has a frequency different from the one of the incident photon due to the lost or gain in energy from a particular vibrational mode of the molecule.

In the Raman spectrum, lower frequency photons give rise to Stokes lines, whereas higher frequency photons are known as Anti-Stokes lines (Figure 2). Usually, the Anti-Stokes lines have small or negligible intensity compared to the Stokes ones, because the population of the excited states is smaller than the one of the fundamental states.

**Figure 3:**
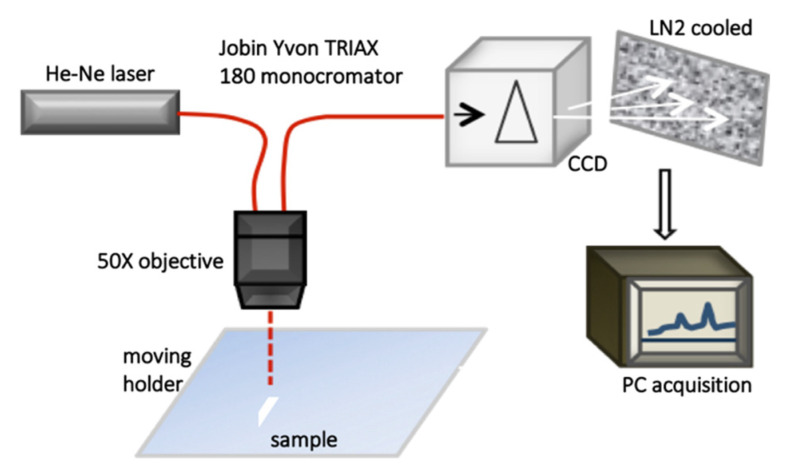
Schematic representation of a micro-Raman spectroscopy experimental setup.

Raman spectroscopy can offer information on the spatial distributions of organic and inorganic compounds in a sample with a spatial resolution of about 1 μm. Furthermore, Raman spectra exhibit little interference with water, making this technique highly convenient for studying many biological specimens, in vivo too. In Figure 3, a schematic representation of a μ-RS experimental setup is reported. This set up has been used for investigating PDL and CGF samples in References [23,25,26,28,29], and it is equipped with a Helium-Neon laser as a light source, coupled to a 50X optical objective. The Raman signal is dispersed by a grating of 1800 grooves/mm and collected by a liquid N_2_ cooled couple charge detector (CCD), then the data are acquired using a dedicated software.

#### 2.3.2. Surface-Enhanced Raman Scattering (SERS)

**Figure 4:**
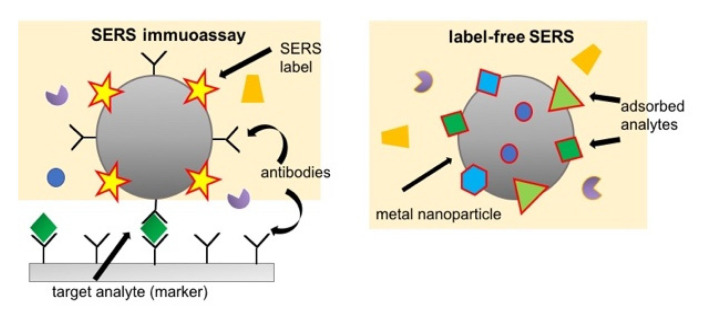
Graphic representation of the two approaches for SERS: the indirect approach (SERS immunoassay) on the left and the direct approach (label-free SERS) on the right.

To enlarge the applicability of Raman techniques on biological tissues and fluids, it is possible to amplify the signal coming from the samples thanks to the Surface Enhanced Raman Spectroscopy (SERS, also named as Surface-Enhanced Raman Scattering). SERS is a vibrational spectroscopy technique that exploits the intensity amplification of Raman signal by metallic nanostructures or metal nanoparticles. At the beginning SERS was adopted to the study of dilute aqueous solutions of a rather simple system such as small molecules or molecular ions, recent nanotechnology, and photonics developments stimulated the application of SERS to more complex bio-systems such as macromolecules, cells, tissues, and bio-fluids. Qualitative and quantitative detection of one or more analytes with SERS can be achieved in a direct or indirect way [31]: in the direct approach (label-free SERS) the spectroscopic signal is due to all those analytes which adsorb on the SERS substrate, whereas in the indirect approach the signal results form a specific SERS label that binds to a target analyte of the sample (see Figure 4). SERS enhancement factor is a very significant parameter in SERS, and it is used for quantifying the overall signal enhancement. To experimentally evaluate SERS enhancement factor measurements of the SERS intensity for the adsorbed molecule on the metal surface, relative to the normal Raman intensity of the “free” molecule in solution are required. The enhancement factor can assume values from 10^10^ to 10^11^, which allows detecting single molecules.

#### 2.3.3. Infrared Spectroscopy

Fourier Transform Infra-Red (FT-IR) spectroscopy is a technique based on the analysis of the spectral IR radiation absorbed by a sample. The energy of IR radiation absorbed corresponds to the energy necessary to the transition between vibrational states of the functional groups. Thus, the spectral intensity of the radiation absorbed by different biochemical bonds within a sample gives rise to the spectrum of IR absorption characteristic, which comprised several peaks or bands, each referred to a particular mode of vibration characteristic of functional groups in the sample [32].

**Figure 5:**
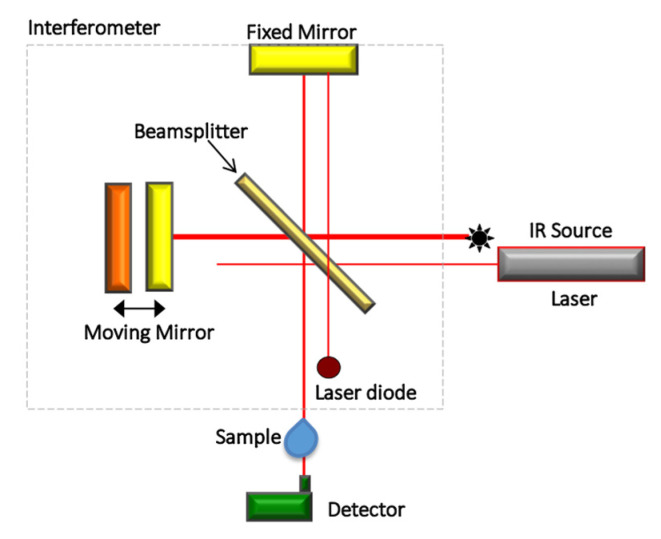
Schematic image of a Fourier-Transform Infrared spectrometer.

The main component of FT-IR set-up is an interferometer (Michelson’s interferometer) which replaces the monochromator of the traditional IR spectrometer model. It is a modern and powerful optical method for collecting absorption spectra in the infrared field by obtaining qualitative information on the structure of the compounds analyzed, as well as, by means of modern software algorithms, also quantitative data. A FT-IR system separates and recombines a light beam leading the recombined light beam to produces an interference pattern dependent on wavelength or interferogram. In particular, the interferometer (Figure 5) consists of a fixed mirror, a moving mirror forming an angle of 90° with the first one, and a beam splitter mirror which is placed at an angle of 45° with respect to the incident beam. The energy of the beam coming from the source is incident on the splitter and comes from this collimated and divided into two perpendicular rays of equal intensity: one reflecting along a path directed to the fixed mirror, and the other passing the splitter and transmitted to the moving mirror. The first ray is reflected by the fixed mirror again on the splitter, from where it is partially returned to the source and partially transmitted and focused on the detector. On the other hand, the moving mirror slides forward parallel to itself along the other branch of the optical path, continuously modifying the distance between it and the splitter, it reflects the other ray on the splitter from different distances and so also this ray is partially returned to the source and partly reflected towards the detector. The energy reaching the detector is therefore the algebraic sum of the two rays, which is switched from the same detector into an electrical signal, which is amplified and represented by an interferogram. The sample to be investigated is placed between the two arms of the interferometer, the frequencies that correspond to the excitation of vibrational states of functional groups of the molecules of the sample are attenuated and the obtained interferogram is converted into the traditional absorption spectrum through the Fourier Transform.

Some practical advantages offered by the use of FT-IR spectroscopy include a minor dispersion of energy, thus, greater energy reaching the detector; time-saving ability to collect spectra in a very short time, since with the FT-IR all frequencies and wavelengths are simultaneously analyzed and recorded by the detector; high precision and accuracy in the discrimination of wavenumbers with the possibility to superimpose the monochrome radiation of a laser source of known frequency as internal standard; finally, the possibility to obtain spectra from a very wide range of samples at any temperature (the source is sufficiently far from the sample and therefore there is no heating effect of the sample) and pressure with appropriate devices and accessories. Moreover, FT-IR spectroscopy can be equipped with a microscope to allow the microscopic analysis of samples or parts of samples up to one micron in size.

#### 2.3.4. Complementarity of Raman and Infrared Spectroscopies

Raman and infrared spectroscopies are chemical analytical methods that provide spectra that contain signals from the functional groups in the sample. Both these techniques are usually coupled to a microscopic approach for biological sample analysis, they are non-destructive, non-invasive, and not time-consuming. These techniques usually require simple or no special preparation procedures for the sample in comparison with the conventional biological methods of analysis. IR measures the light absorption by specific molecules using a broadband light source, the Raman technique measures the characteristics of Raman emission induced from molecules under monochromatic laser irradiation. It is important to underline that FT-IR and Raman spectroscopy are complementary techniques because the bands in FT-IR spectra are due to polar functional groups while the bands in Raman spectra are due to non-polar functional groups. Thus, the use of both the described vibrational spectroscopies is suggested to detect all the qualitative and quantitative information of the samples included in the study.

### 2.4. Data Analysis

The signal intensity of the spectroscopy response of biomaterials is usually relatively weak, especially in the case of Raman spectroscopy. Moreover, fluorescence mechanisms can often occur affecting the insight of spectral data. Numerical data treatments are generally used for subtracting background spurious signal and decrease the uncorrelated noise component [33,34,35]. Numerical data processing for removing background signals from the Raman spectra are exhaustively reviewed in Reference [36]. A numerical filtering process based on wavelet algorithms has been also developed for both remove the background signal and improve the signal/noise level [23,24,26,37]. By using special scaled functions, named “wavelets”, the method allows a decomposition of the experimental spectrum in components with increasing spectral detail, the provides the basis of a filter bank algorithm able to attenuate both high-frequency signal components (noise) and low-frequency components (smooth background signal). The detail of the method implementation is reported in Camerlingo et al. 2006 [37].

A normalization of the data, with respect to the signal intensity, is also demanding to compare different spectra. Different criteria can be used for this purpose, choosing as normalization reference the intensity of a fixed mode [24] or the total area of the spectral signal or the square standard deviation of data, with respect to the mean intensity value [23,26,28].

Similarities between spectra acquired from analogous samples in equivalent experimental conditions, and differences between spectra from samples collected in different stages of orthodontic treatment can be evidenced by a univariate analysis. A comparison between the intensity of one value of a spectrum and the corresponding value of another spectrum collected from another sample was performed by using a linear regression or univariate analysis of data. This analysis offers a numerical coefficient that spans from 0 for uncorrelated data, to 1 for perfect linear dependence [27].

The deconvolution of the spectrum in elemental components allows to individuate the main modes occurring. Typically, Lorentzian functions were used for modeling Raman spectra, while Gaussian and mixed Gaussian-Lorentzian functions are more indicated in the case of FT-IR spectra and for broad Raman mode components. Their spectral positions, intensities, and widths were evaluated by a fitting procedure. In some case, mainly for FT-IR data, the second derivative of the spectrum is also performed to preliminarily evaluate the local minima which correspond to the vibrational modes [38].

For classification of Raman spectra from PDL sample a multivariate analysis was also adopted. Principal component analysis (PCA) is particularly adequate for analyzing Raman spectra of complex biological samples. The spectral data are decomposed by using a mathematical procedure that decreases the data dimensions to a smaller number of scores and principal components (PC) that give the most important information of the spectra. PCA can be performed not on all the investigated spectral region but only on properly selected intervals, in this case interval-PCA (i-PCA) is executed [26].

## 3. Results and Discussion

In five of the selected studies, samples were collected before and after 2, 7, and 14 days of OTM [23,26,27,28], in one after 1, 7, and 28 days [24] and in another only after 28 days [25]. The main Raman modes were identified by the centers of the Lorentzian components evaluated by fitting the spectroscopy data [39] and are reported in Table 2 for PDL and in Table 3 for GCF samples, together with their assignments.

### 3.1. Periodontal Ligament Analysis

#### 3.1.1. μ-RS Results

**Figure 6:**
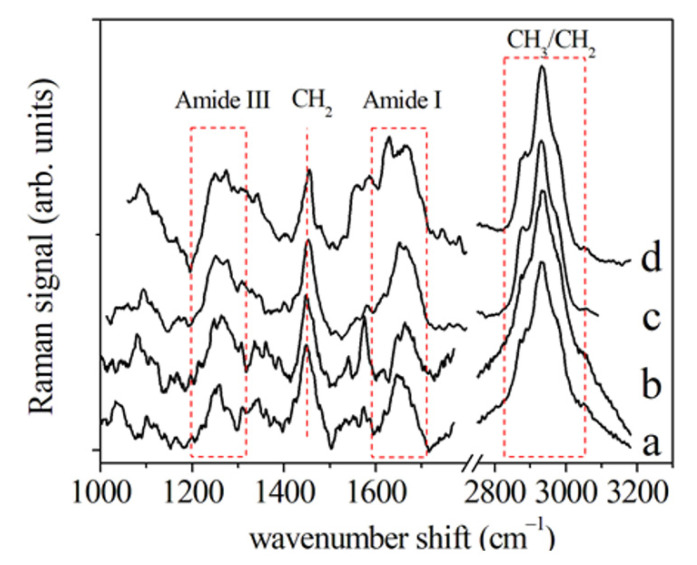
Raman spectra of PDL samples from premolars extracted (**a**) before, (**b**) after 2 days, (**c**) 7, and (**d**) 14 days of orthodontic treatment. Protein and CH_2_/CH_3_ contributions were indicated. (Reprinted with permission from Perillo et al., 2020 under Open Access conditions).

Camerlingo et al. 2014 [23] and Perillo et al. 2020 [28] assessed the changes of PDL from premolars that were extracted from patients under OTM by using the difference observed in Raman spectra collected after 2, 7, and 14 days of orthodontic treatment. In Figure 6, a representative Raman spectrum (a) obtained by averaging all the spectra from the control PDL samples is reported together with the average spectra of the PDL samples after 2 (b), 7 (c), and 14 days (d) of in vivo ~0.5 N force application. In these spectra, the regions related to Amide I and CH_3_/CH_2_ bands were prevalent. The contributions at 1600–1700 cm^−1^ (Amide I mode) and 1200–1300 cm^−1^ (Amide III mode) were attributed to protein vibrations and the broadband at 2930 cm^−1^ was due to the CH_2_/CH_3_ bond vibrations. A further Raman mode, at about 1450 cm^−1^ is characteristic of CH_2_ scissoring Raman mode and protein components.

**Figure 7:**
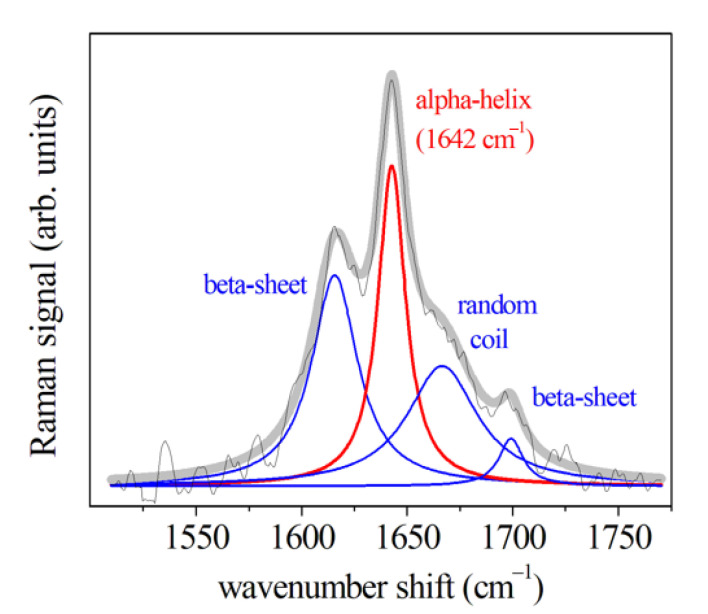
Deconvolution of the average Raman spectrum of PDL in the region of Amide I. This spectrum was evaluated by averaging the Raman spectra of samples collected before OTM. Red land blue lines indicate the α-helix component and the other main components of the Raman band, respectively. (Reprinted with permission from Perillo et al., 2020 under Open Access conditions).

The Amide I spectral region of a PDL control sample (Figure 7) presented different components that are usually correlated to the secondary structure of the protein. More in detail, the mode at 1645–1650 cm^−1^ was assigned to the α-helix component dominated the band. The further peaks at about 1620, 1668, and 1680 cm^−1^ were related to β-sheet or collagen 3_10_-helix, β-turn, and β-sheet secondary structure conformations, respectively (see Table 2).

By comparing the Amide I regions of spectra from samples collected at different times, it was possible to notice many changes in the peak centers (in cm^−1^) of the modes above mentioned (see Table 2). In particular, the intensity of the α-helix Raman mode and the whole Amide I band lowered with respect to the same contributions in control PDL Raman spectra [23,28]. The other components of Amide I band enlarged and increased their intensity in comparison with the α-helix mode. To note, the α-helix mode can be considered as a hierarchical structure in which are present H–bonds at the higher molecular level originated from individual hydrogen (H) bonds. The α-helix is characterized a single-stranded conformation with a spring-like protein structure with 3-4 H–bonds per turn. In the case of collagen, a particular triple helix configuration is considered for α-helix. This structural arrangement is the most effective bond arrangement for ensuring the thermodynamic and mechanical stability together with high elasticity and large deformation capacity. A partially unfolded α-helix structure with a combination of broken and intact H–bonds along the filament axis can be associated to the β-sheet structure. The most relevant changes were observed in Raman spectra after 48 h of tooth movement while they were quite recovered on day 7 of PDL collection. When a strain is applied to the tissue, a stress necessary for the beginning of a modification of the α-helix structure or transformation from the α-helix to the β-sheet conformation, is caused by the above-discussed unfolding-refolding processes, that induces a hydrogen relocation, and an increase in disorder [28].

**Figure 8:**
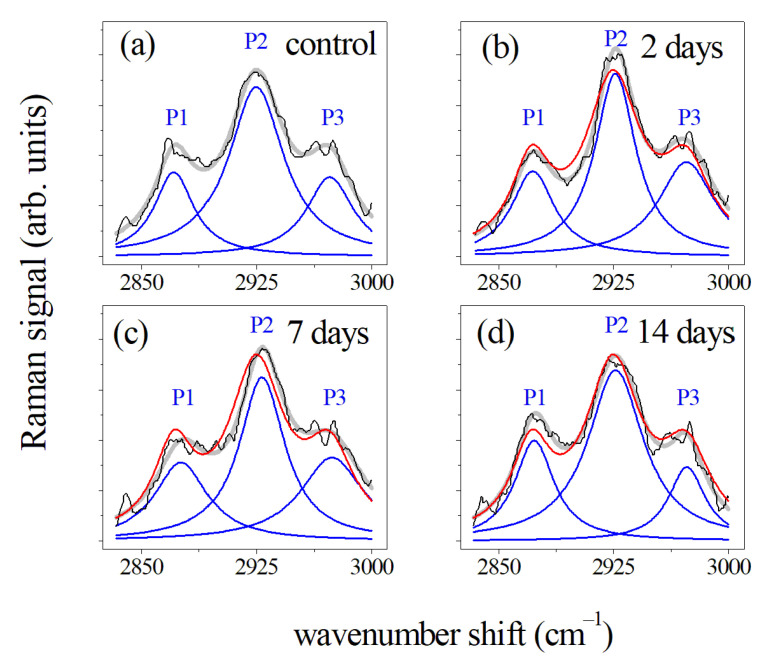
Deconvolution of the average Raman spectra of PDL samples collected before (**a**) and after (**b**) 2, (**c**) 7, and (**d**) 14 days of OTM in the 2800–3000 cm^−1^ spectral region. Black lines indicated the measured spectra and blue lines are related to the main P1, P2, and P3 Lorentzian components of the Raman band. Fit of the experimental data resulting from the deconvolution process are indicated by gray lines. All the band areas were normalized to 1. In the (**b**–**d**) panels the spectra were compared with the Raman response (red line) of the control of (**a**) panel. (Reprinted with permission from Perillo et al., 2020 under Open Access conditions).

The Raman spectra of the CH_3_/CH_2_ modes for PDL samples collected before (a), and after 2 (b), 7 (c), and 14 days (d) of the orthodontic force application are reported in Figure 8.

These average spectra were normalized according to the procedure previously described and presented a large and predominant peak at 2930 cm^−1^. This feature is typically attributed to CH_3_ symmetric stretching vibration. The other two peaks located at 2875 cm^−1^ (P1) and 2970 cm^−1^ (P3), were also assigned to C–H bond modes, in particular, to CH_2_ asymmetric and CH_3_ asymmetric stretching modes, respectively [28,38]. The quantitative analysis of these data (see Reference [28]) shown that the intensity of P1 and P2 peaks initially increased after 2 days of force application, then decreased after 7 days of treatment and after manifested an increase after two weeks of force application. A different behavior was noticed for the P3 peak after 14 days of OTM.

#### 3.1.2. FT-IR Results

**Figure 9:**
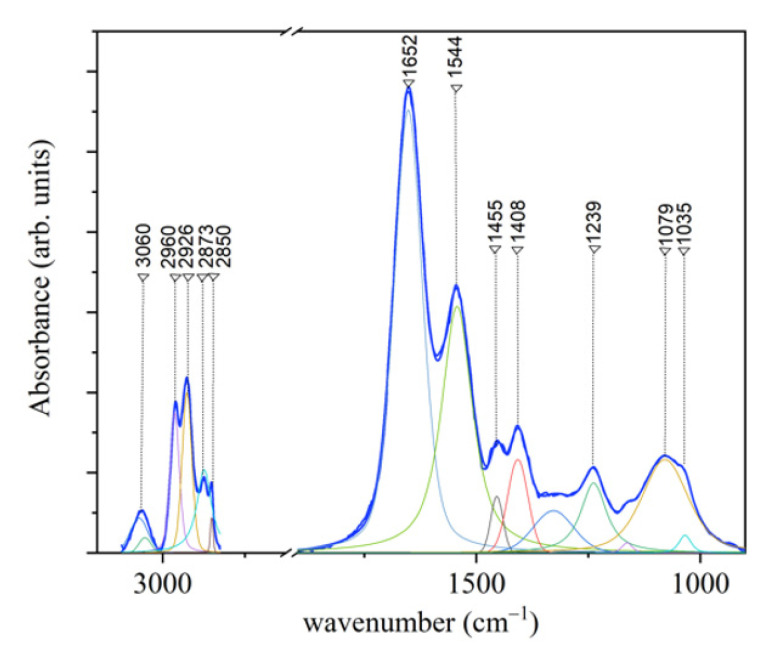
Representative FT-IR spectrum of PDL in the 3100–1000 cm^−1^ with the deconvolution analysis of peaks with Lorentzian–Gaussian curves (blue line: experimental curve). (Reprinted with permission from Camerlingo et al., 2020).

In Figure 9, an average infrared spectrum acquired from PDL samples collected before OTM is reported. In this Figure also the results of the deconvolution procedure performed by using Lorentzian–Gaussian curves is shown.

**Figure 10:**
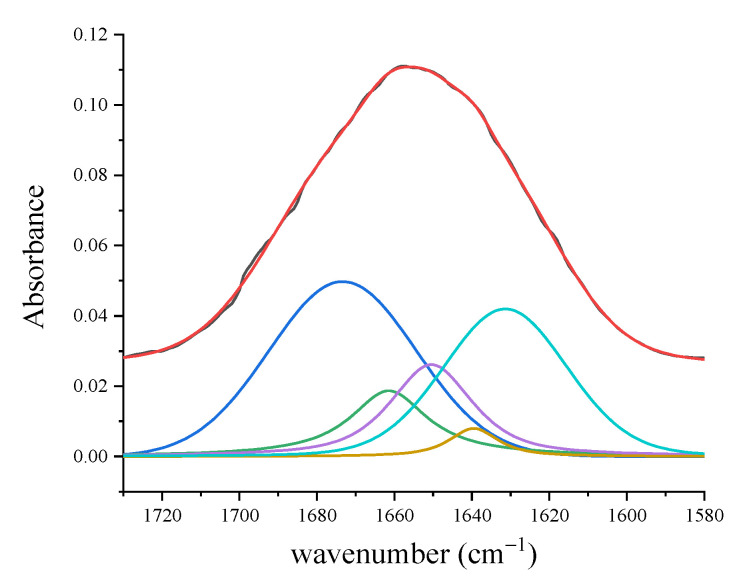
Spectrum of PDL in the Amide I region (1710–1580 cm^−1^) (black line). Colored lines indicate the different contributions obtained by the deconvolution analysis of peaks using Lorentzian–Gaussian curves. Fit of the experimental data resulting from the deconvolution process is indicated by the red line. (Reprinted with permission from Portaccio et al., 2019).

The main features of the spectrum and the related assignments are reported in Table 2. The region associated to C–H bonds in the region 3100–2800 cm^−1^ is mainly related to collagen contribution that has been also evidenced by SEM micrographs [29] and to a lesser extent to lipid content. In the 1750 to 1000 cm^−1^ spectral range, the band at 1656 cm^−1^ is related to the C=O stretching of Amide I (Table 2). By evaluating the subcomponents of the Amide I band, a characterization of the secondary structure of protein component can be obtained. As shown in Figure 10, the deconvolution of this region (1750–1580 cm^−1^) evidences the presence of different subcomponents. The contribution at 1673 cm^−1^ is attributed to β-turn subcomponent, the ones at 1661 and 1650 cm^−1^ are assigned to the α-helix subcomponent, and the features at 1639 and 1631 cm^−1^ are due to β-sheets contributions.

### 3.2. Gingival Crevicular Fluid Analysis

#### 3.2.1. SERS Results

**Figure 11:**
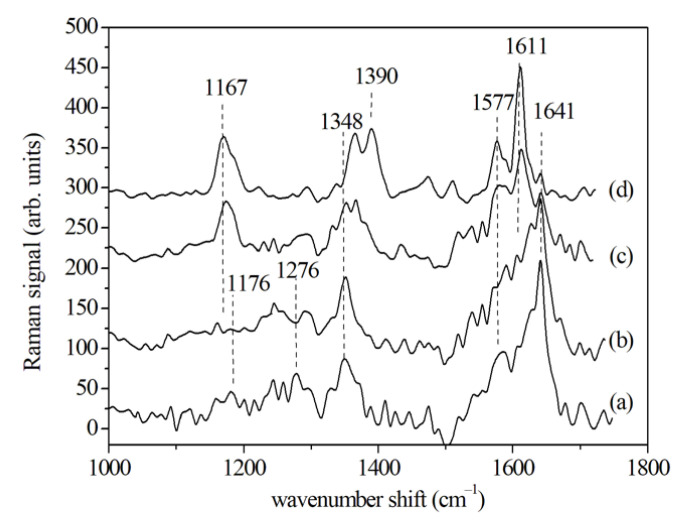
SERS spectra of GCF samples collected before (T0), after 2 (T1), after 7 (T2), and 14 days (T3) the beginning of the OTM. For facilitating the visual inspection, the spectra are arbitrarily shifted along the vertical axis. (Reprinted with permission from d’Apuzzo et al., 2017).

Typical SERS spectra with the principal vibrational modes, obtained from GCF samples in the 1000 to 1800 cm^−1^ range before (T0) and after (T1, T2 and T3) the application of an orthodontic force, are reported in Figure 11. The attention was focused on 1000 to 1700 cm^−1^ wavenumber range and on the five main modes at about 1176, 1276, 1348, 1577, and 1641 cm^−1^. The mode at 1176 cm^−1^ can be attributed to the nucleic acid base C–N. The peaks at 1242 and 1276 cm^−1^ can be due to different components of Amide III (α-helix or β-sheet) and the 1276 cm^−1^ peak may also indicate a contribution from CH_2_ deformation mode. The structure present at 1348 cm^−1^ can be attributed to adenine and guanine of nucleic acids. The small peak at 1470 cm^−1^ is due to CH_2_, the one at 1577 cm^−1^ which can be related to Amide II and cytochrome, and the large structure at 1641 cm^−1^ is typical of protein spectra and is related to Amide I (see Table 3). As done for PDL, GCF samples from untreated case (T0) and after 2 (T1), 7 (T2) and 14 (T3) days of OTM have been considered. From T0 to T1 of OTM, the intensity of Raman mode at 1276 cm^−1^ decreases, whereas the Amide I band intensity increases in T1 and decreases in the subsequent T2 and T3 stages (see Figure 11). This behavior can be ascribed to changes in protein concentration.

**Figure 12:**
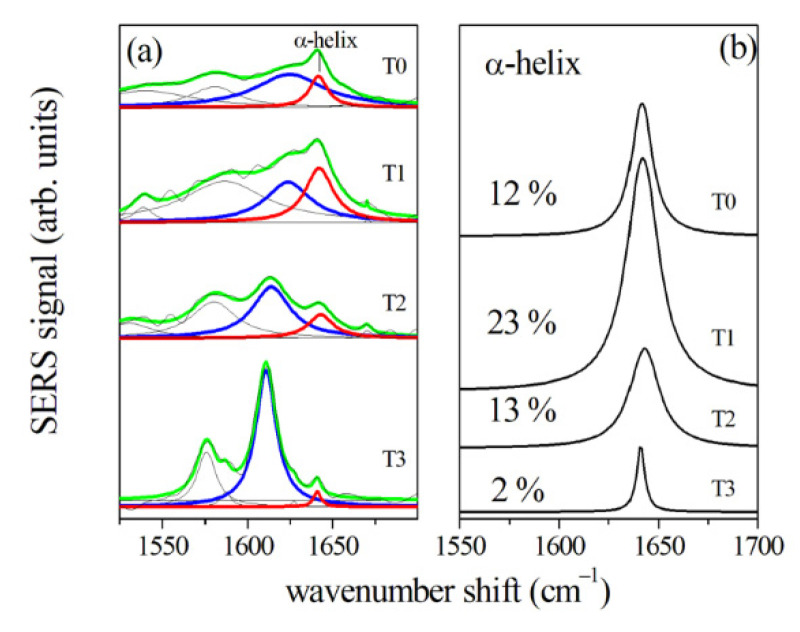
(**a**) Amide I region of SERS spectra of GCF samples collected from an 18-year-old patient before (T0) the beginning of the OTM, (T1), (T2), and (T3) refer to samples collected after 2, 7, and 14 days after, respectively. Black lines indicate the experimental data that are fitted by a convolution of Lorentzian peaks indicated by green lines. The main components are also plotted using colored lines. Red and blue lines indicate the α-helix and the β-sheet components, respectively. (**b**) The α-helix component at T0, T1, T2, and T3 stages. Percentage value of the α-helix area normalized to the whole amide I band area is reported for each plot. (Reprinted with permission from d’Apuzzo et al., 2017).

More detailed information can be obtained also in this case from the Amide I band deconvolution (Figure 12). During the OTM, the center of the Amide I band moves toward shorter wavenumber shifts, specifically for T2 and T3 spectra. These changes are related to the decrease in the α-helix subcomponent (located around 1640 cm^−1^) and the increase in the β-sheet subcomponent (located in the range between 1610 and 1625 cm^−1^). The percentage of α-helix subcomponent to the Amide I band was evaluated by estimating the ratio of areas of the α-helix peak and the whole Amide I band, and it is shown in Figure 12b for the different stages of the OTM. The present results of the

Amide I contributions at the different stages are not in complete agreement with the results reported by Jung et al. [24]. This could be ascribed to different modalities in GCF sampling and to the different days of collection.

#### 3.2.2. μ-RS Results

**Figure 13:**
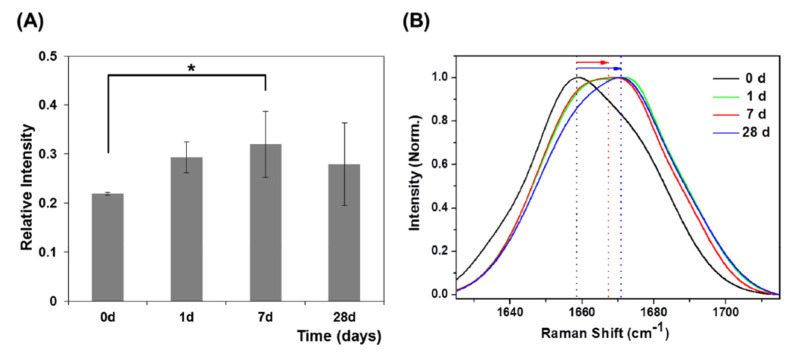
(**A**) Relative intensities in 1667 cm^−1^ Raman peaks during OTM. * Significantly different from day 0 (*p* < 0.05, one-way ANOVA with Turkey’s Honest Significance Difference (HSD) post-hoc procedure). (**B**) Normalized average GCF Raman spectra in the 1625–1715 cm^−1^ range during OTM. (Reprinted from Jung et al. 2014 under Open Access conditions).

The μ-Raman analysis was performed on the GCF paper points without any sample manipulation in order to develop an effective approach for monitoring the processes occurring during OTMs. The Raman spectra of the GCF collected using paper cones showed similar features observed in the SERS spectrum above (see Table 3). Much information was obtained by analyzing the 1500 to 1750 cm^−1^ region where the Amide I band is located. In agreement with the SERS results, also μ-RS results show that the center of the band moved toward shorter wavenumber shifts in the days after the beginning of the treatment. As said before these results are not in complete agreement with the outcomes reported by Jung et al. [24] (see Figure 13). This discrepancy cold be due to different modalities in experimental procedure.

**Figure 14:**
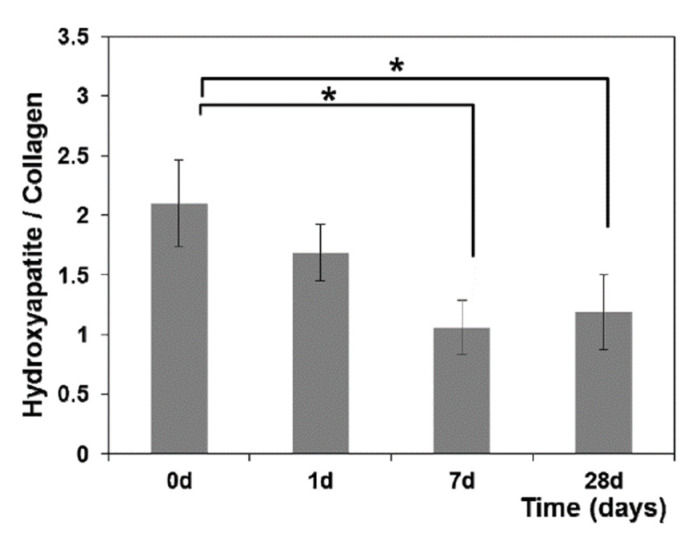
Hydroxyapatite to collagen ratio in GCF during OTM. * Significantly different from day 0 (*p* < 0.05, one-way ANOVA with Turkey’s HSD post-hoc procedure). (Reprinted from Jung et al., 2014 under Open Access conditions).

Jung et al. [24] reported also the results of a ratiometric analysis of Raman spectra collected at different times after starting OTM. As an example, in Figure 14 the intensity ratio between the hydroxyapatite to primarily collagen-dominated matrix band is reported. The values of this ratio indicate significant changes in the amount of mineralization. In particular, hydroxyapatite and collagen ratios decreased at day 7 (*p* < 0.05), indicating a decrease in the mineralization due to a reduction in hydroxyapatite content during the alveolar bone remodeling process. Other intensity ratios able to give interesting information are the ones related to carbonate/Amide I and carbonate apatite/hydroxyapatite bands, also reported in Reference [24].

To calculate the percentage of success in matching peak assignments in Raman spectra with the OTM time points, a multivariate analysis (i-PCA) showed that the OTM stage was exactly recovered in 98% of Raman spectra with 100% of correct assignment for T0 and T2 spectra and 92% of T1 spectra. Some T1 spectra were incorrectly assigned as T0 [26]. Thus, the use of Raman spectroscopy and multivariate analysis of GCF spectra can enable to recognize the exact time point of the OTM. This innovative approach might be useful for the clinician for choosing the type of treatment for each patient and speed up and make progress in the orthodontic therapy.

#### 3.2.3. FT-IR Results

**Figure 15:**
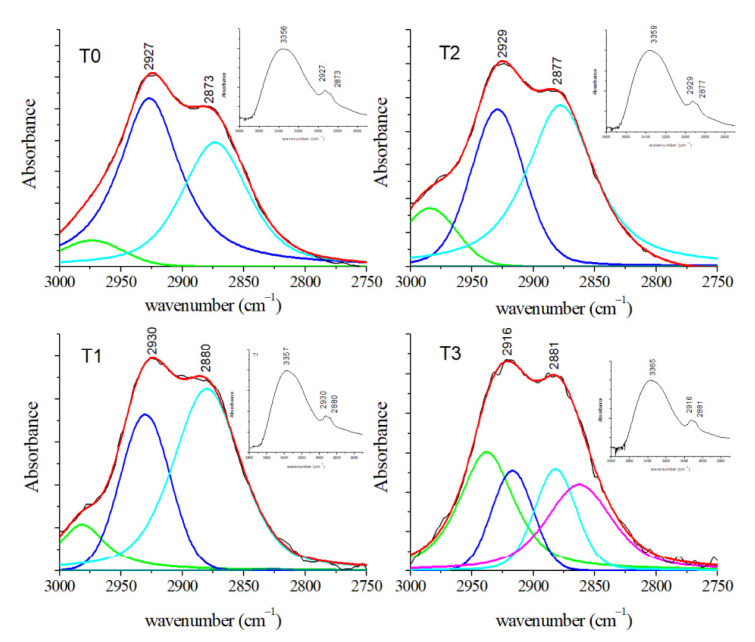
Average FT-IR spectra of GCF samples obtained from patients before (T0) and during OTM in the 3000–2750 cm^−1^ spectral region (black lines). The deconvolution analysis of peaks with Gaussian–Lorentzian curves is shown by colored lines. Red lines indicated the fitted spectra. (Reprinted with permission from Portaccio et al., 2019).

**Figure 16:**
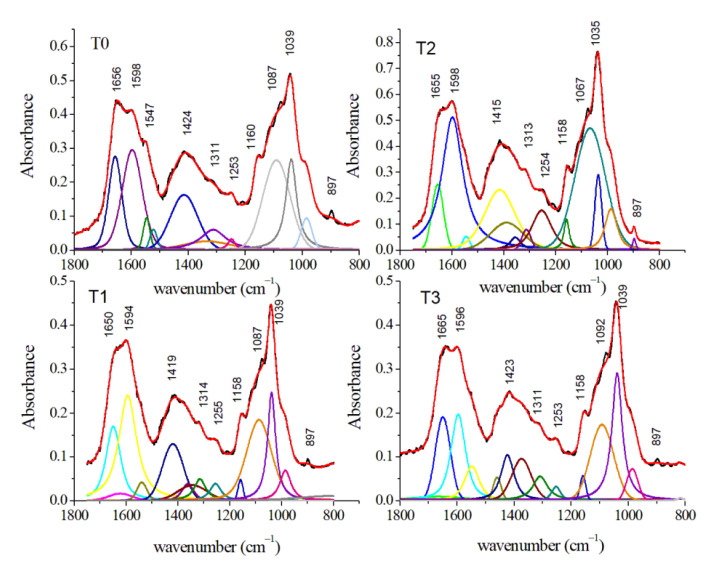
Average FT-IR spectra of GCF samples obtained from patients before (T0) and during OTM in the 1800–800 cm^−1^ spectral region (black lines). The deconvolution analysis of peaks with Gaussian-Lorentzian curves is shown by colored lines. Red lines indicated the fitted spectra. (Reprinted with permission from Portaccio et al., 2019).

FT-IR spectra were preliminarily analyzed using the univariate analysis briefly described in Section 2.3. Such univariate analysis was used to evaluate any correspondences in spectra from samples in the similar experimental conditions and any differences between spectra from GCF samples at different OTM timepoints [27]. The average spectra between 3800 and 800 cm^−1^ for GCF samples before (T0) and after 2 (T1), 7 (T2), and 14 (T3) days of orthodontic treatment are reported in Figure 15 and Figure 16, respectively.

The 3000–2800 cm^−1^ region is particularly relevant due to the presence of different features related to lipid contents. GCF samples at T1, T2, and T3 showed several modifications due to shifts of the different contributions over time (Figure 15). In particular, the peaks at 2927 and 2870 cm^−1^ can be assigned to CH_2_ antisymmetric and CH_3_ symmetric stretching, respectively (see Table 3). Thanks to the deconvolution procedure it is possible to evidence a third small component centered at 2974 cm^−1^ that can be due to antisymmetric CH_3_ stretching mode of lipids. The average FT-IR spectra in the 1750–800 cm^−1^ region for GCF samples at different stages of OTM were reported in Figure 16. Visual inspection of the spectra evidenced the presence of some relevant contributions. In T0 spectrum, the band at 1656 cm^−1^ is assigned to the C–O stretching of Amide I. The peak at 1598 cm^−1^ can be assigned to C=C stretching of amino acids. Other two important peaks are located at 1424 and 1039 cm^−1^ and they can be ascribed to COO- stretching of amino acids and to C–O stretching of glucose, respectively. In addition, some small contributions are positioned at 1311, 1160, and 897 cm^−1^ and they are assigned to CH_2_ twist of lipids and amino acids, antisymmetric C–O stretching and COH_2_ bending of lipids, and C–O stretching of glucose, respectively. Analogous contributions are also evident in the spectra of GCF samples collected in T1, T2, and T3 stages. The above-described deconvolution procedure permits to evidence other spectral features in Figure 16. This analysis for T0 spectrum shows other contributions at 1547 cm^−1^ due to the N–H bending of Amide II and at 1376 cm^−1^ due to symmetric bending of CH_3_ of nucleic acids. The deconvolution procedure manifests also the presence of features located at 1253 and 1087 cm^−1^ ascribed to antisymmetric and symmetric PO_2_^−^ stretching of nucleic acids, respectively. At 984 cm^−1^ is also present a contribution due to C–O and C=C stretching mode of amino acids. Moreover, in this region, some peaks are characterized by a wavenumber shift. For Amide I and II, these shifts are a signature of changes in the protein secondary structure. In Table 3, the spectral features present in the wavenumber regions here examined are reported for the different stages of the OTM, together with their assignments. Conformational changes and rearrangements of existing proteins, lipids, and nucleic acid structures are evidenced by the above-mentioned changes in the FT-IR spectra according to expectances because OTM induces inflammatory processes and changes in several pro-inflammatory cytokines (i.e., IL-6, IL-1, IL-1ß) enzymes (i.e., alkaline/acid phosphatase), and metabolites (i.e., prostaglandins E2) concentrations [2,40]. The results obtained by FT-IR spectroscopy agree also with the ones obtained by Raman spectroscopies [23,24,27].

## 4. Conclusions and Implications for Research and Practice

The vibrational spectroscopies described in this research can be considered the techniques of choice in comparison with other methodologies thanks to the ability to monitor vibrational contributions from the molecules in biofluids and fibers. Thus, it is possible to assert the innovative potential role that Raman and FT-IR spectroscopies can play in orthodontics, but more generally in dental sciences, in the evaluation and monitoring of periodontium during different clinical conditions. The analyses of GCF and PDL samples through the vibrational spectra showed similar results in terms of biochemical changes over time during the first weeks of OTM, as demonstrated by previous studies using other analytical techniques. In particular, the analysis of μ-RS directly performed on paper points impregnated of GCF, easily and quickly collected during the orthodontic appointment, is a potential appealing methodology, noticeably less expensive and less laboratory-based in comparison to other analytical techniques described in the literature.

Limitations of this study are the inclusion of a limited number of studies and a small number of patients (a total of about 63) that may be enlarged to get more evidence. Thus, the work until done in this field may be considered as a starting point for future prospective cohort studies with a wider sample using different typologies of orthodontic appliances (i.e., removable vs. fixed) assessed with vibrational spectroscopies and comparing the outcomes with other analytical methodologies and a greater interest in these optical techniques would be beneficial for dental and orthodontic research field.

This research field could be a novel step for the development, in the near future, of a vibrational spectroscopy-based non-invasive, accurate, and reliable test. Clinical implications may be found both for diagnosis and clinical purposes in specific type of patients presenting different pathological conditions (i.e., bony defects, arthritis) before starting treatment and for monitoring the status of periodontal tissues during orthodontic tooth movement by GCF collection repeated overtime. This may help the clinician in the choice of the proper type of force application on teeth individually for each patient with the final aim of improving treatment outcomes and even accelerating its overall duration.

## Figures and Tables

**Table 1:** Characteristics of the selected studies.

Author, Year [Ref. n°]	Vibrational Spectroscopy	Sample Type	OTM Time (Days)	Subject Number	Age (yrs)
Camerlingo et al., 2014 [23]	μ-RS	PDL	0, 2, 7, 14	3	range 13–21
Jung et al., 2014 [24]	μ-RS	GCF	0, 1, 7, 28	10	range 18–23 mean 20.8 ± 2.5
Camerlingo et al., 2015 [25]	μ-RS	GCF	0/28	3	range 13–26
D’Apuzzo et al., 2017 [26]	μ-RS/SERS	GCF	0, 2, 7, 14	18	range 13–22
Portaccio et al., 2019 [27]	FT-IR	GCF	0, 2, 7, 14	18	range 12–22
Perillo et al., 2020 [28]	μ-RS	PDL	0, 2, 7, 14	11	range 11–24 mean 19.9 ± 4.7
Camerlingo et al., 2020 [29]	μ-RS/FT-IR	PDL	0	3	range 13–22

**Table 2:** μ-RS and FT-IR main peak assignments for PDL samples References [23,28,29,39].

Assignment Mode	Peak Position Raman (cm^−1^)				Peak Position FT-IR (cm^−1^)			
	0	2	7	14	0	2	7	14
PO_2_^−^ asymmetric stretching C-O-P stretching					1239			
Amide III (β-sheet)	1243	1245	1247	1252				
Amide III (random coil)	1258	1265	1266	1273				
Amide III (α-helix)	1309	1307	1310	1307				
CH_2_	1450				1455			
Amide II					1544			
Amide I (3_10_-helix; β-sheet)	1617	1619	1618	1621	1631			
Amide I (α-helix)	1642	1643	1641	1642	1650			
Amide I (β-turn)	1668	1662	1661	1666	1661			
Amide I (β-sheet)	1695	1687	1690	1695	1673			
CH_2_ asymmetric stretching	2875				2926			
CH_3_ symmetric stretching	2930				2873			
CH_3_ asymmetric stretching	2970				2960			

**Table 3:** μ-RS, SERS, and FT-IR spectroscopy peak position and assignments for GCF samples References [24,25,26,27,39].

Assignment Mode	Peak Position SERS (cm^−1^)				Peak Position Raman (cm^−1^)				Peak Position FT-IR (cm^−1^)			
	0	2	7	14	0	2	7	14	0	2	7	14
S–S bond stretching	465											
Phenylalanine	621											
Tyrosine	825											
Deoxyribose bending CO_2_H of tyrosine	895								897			
PO_4_^3−^	946											
PO_4_^3−^ ν1 symmetric stretching, apatite					984	985	986	986				
C–H bending phenylalanine	1007				1002							
C–O stretching of carbohydrates									1039	1038	1035	1039
symmetric PO_2_^−^ stretching of nucleic acids									1087	1087	1067	1092
Nucleic acid base PO_2_^−^						1100	1100					
C–O asymmetric stretching and COH bending of lipids									1160	1158	1158	1158
Cytochrome						1167	1167	1167				
Nucleic acid C–N	1176				1176							
Amide III	1242	1242	1242									
PO_2_^−^ asymmetric stretching of nucleic acids									1253	1255	1254	1253
Amide III, CH_2_ deformation	1276				1280							
CH_2_/CH_3_ twisted									1311	1314	1313	1308
Adenine/guanine of nucleic acids	1348	1348	1348		1345	1345	1345					
symmetric bending of CH_3_ of nucleic acids									1376	1361	1388	1384
Cytochrome			1390									
COO– stretching of aminoacids									1424	1419	1415	1423
CH_3_ symmetric stretching CH_2_ lipids/proteins scissoring										1462	1459	
CH_2_	1470											
Carotene						1540	1540					
Amide II (N–H bending of proteins)									1547	1539	1545	1548
Amide II/cytochrome	1577				1575	1577	1577	1577				
C=C stretching of amino acids									1598	1594	1598	1596
Amide I (β-sheet)					1625	1625	1611					
Amide I (α-helix)	1641				1640	1640						
C=O stretching of proteins (Amide I band)									1656	1650	1655	1655
CH_3_ symmetric stretching of lipids									2873	2880	2877	2881
CH_2_ asymmetric stretching of lipids									2927	2930	2929	2916
CH_3_ asymmetric stretching of lipids									2974	2981	2984	2937
NH stretching of Amide A OH stretching									3356	3357	3359	3365

## Data Availability

The data presented in this study are available on request from the corresponding author.
